# An ectopic adrenocortical oncocytic adenoma in the liver highly mimicking hepatocellular carcinoma: case report and literature review

**DOI:** 10.1186/s13000-021-01097-0

**Published:** 2021-07-04

**Authors:** Jingci Chen, Xueshuai Wan, Yao Lu, Wenze Wang, Dachun Zhao, Zhaohui Lu, Yilei Mao, Jie Chen

**Affiliations:** 1 Department of Pathology, Peking Union Medical College Hospital, Chinese Academy of Medical Sciences and Peking Union Medical College, 100730 Beijing, China; 2 Department of Liver Surgery, Peking Union Medical College Hospital, Chinese Academy of Medical Sciences and Peking Union Medical College, 100730 Beijing, China

**Keywords:** Ectopic, Intrahepatic adrenocortical adenoma, Oncocytic, HCC, Case report

## Abstract

**Background:**

Ectopic adrenocortical tissue is a lesion usually found incidentally during autopsy or inguinal surgery. Here, we demonstrate an extremely unusual case of intrahepatic adrenocortical adenoma which highly mimicks hepatocellular carcinoma (HCC) and brings challenges for clinicians and pathologists. The diagnostic pitfalls have been discussed in detail to provide clues for guiding differential diagnosis and future treatment.

**Case presentation:**

A 44-year-old man was admitted into our hospital for evaluation of a hepatic mass identified during routine examination. Enhanced CT revealed its margin displayed apparent enhancement in arterial phase, but hypointensity in portal and delayed phase. HCC was suspected and partial hepatectomy was performed. Microscopically, cells were arranged in solid sheets. Most of the tumor cells were large, polygonal, had prominent nucleoli and were rich in eosinophilic cytoplasm. Pleomorphic nucleus was frequently found. Focally, smaller cells were found with small nuclei and granular cytoplasm. Immunohistochemically, tumor cells were negative for Arg-1, glypican-3 (GPC3), hepatocyte specific antigen (HSA), and positive for synaptophysin (Syn), α-inhibin, and Melan A. The Ki-67 index was 1 %. The final diagnosis was ectopic adrenocortical oncocytic adenoma and the patient was uneventful after the surgery.

**Conclusion:**

Intrahepatic adrenocortical adenoma in the liver can hardly be diagnosed through radiology and little experience in pathology has been reported. In the present case, massive oncocytic changes and huge pleomorphism add greatly to the difficulties of making correct diagnosis. This lesion should be carefully kept in mind and a combination of markers is suggested for differentiating from HCC.

## Introduction

Ectopic adrenocortical tissue is a condition where adrenocortical tissue appears in places other than the adrenal glands. The ectopic tissue can also form neoplasms such as adenoma or carcinoma [1]. Embryologically, the adrenal cortex derives from coelomic mesoderm, with its neighboring structures including celiac plexus, renal parenchyma, broad ligaments, spermatic cord, and testis [2]. Therefore, typical sites for ectopic adrenocortical tissue or neoplasms are the renal hilum, retroperitoneum, and spermatic cord [3]. Most ectopic adrenocortical tissue or neoplasms are found in neonates, and they occur much less commonly in adults due to atrophy [4]. Based on its mostly benign characteristic, surgical removal is the main treatment. The purpose of preoperative diagnosis is to localize it and differentiate it from malignant lesions. Pathologically, it can usually be differentiated from primary lesions of the ectopic sites based on the classic growth pattern and cells. Here, we present an extremely rare case of ectopic adrenal cortical oncocytic adenoma occurring in the liver in a 44-year-old man, which might be easily misdiagnosed as primary hepatocellular carcinoma (HCC).

## Case presentation

Clinical history.

**Fig. 1:**
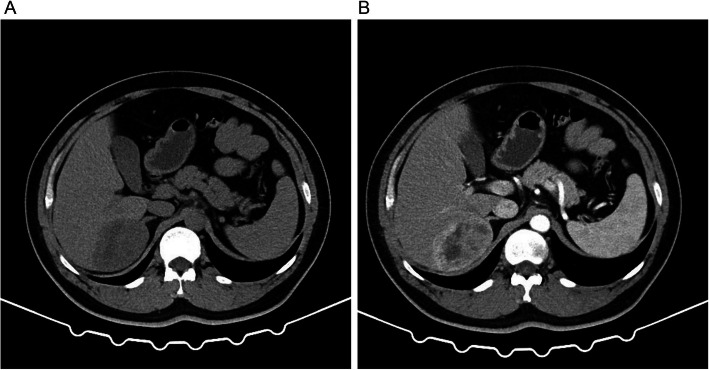
CT scanning of the mass. **a** A large mass was located between segment 6 of the liver and right adrenal gland. **b** In enhanced CT scanning, the margin of the mass displayed apparent enhancement in arterial phase

A 44-year-old man was admitted into our hospital for evaluation of a hepatic mass in April 2020. The mass was first discovered during regular examination in a local hospital five years ago. Ultrasound showed a 4.24 × 2.63 cm hypoechoic nodule in the right lobe of the liver. Abdominal computed tomography (CT) revealed a well-circumscribed oval mass with low density in segment 6 of the liver. Enhanced CT indicated that the mass was heterogenous with obvious enhancement in the margin and a hemangioma was suspected. The patient underwent regular examination in the following four years and the mass was growing slowly. In January 2020, enhanced CT revealed a much larger mass with the maximum diameter of 7.8 cm in the space between segment 6 of the liver and right adrenal gland (Fig. 1). The margin displayed apparent enhancement in arterial phase, but hypointensity in portal and delayed phase compared with the surrounding liver tissue. A primary liver malignancy was suspected by clinicians. During the follow-up time, the patient was asymptomatic. In blood tests, the patient was negative for serum hepatitis B surface antigen and anti-hepatitis C virus, and was positive for hepatitis B surface antibody. Tumor markers including α-fetoprotein, carcinoembryonic antigen, and carbohydrate antigen 199 were all within normal range. The patient had no smoking, drinking, medical, or psycho-social history. No genetic tests were performed previously. Physical examination revealed no obvious abnormalities.

### Operation

The mass was located on the border of segment 6 and segment 7 of the liver and was close to the inferior right hepatic vein. Partial hepatectomy was performed.

#### Pathology

**Fig. 2:**
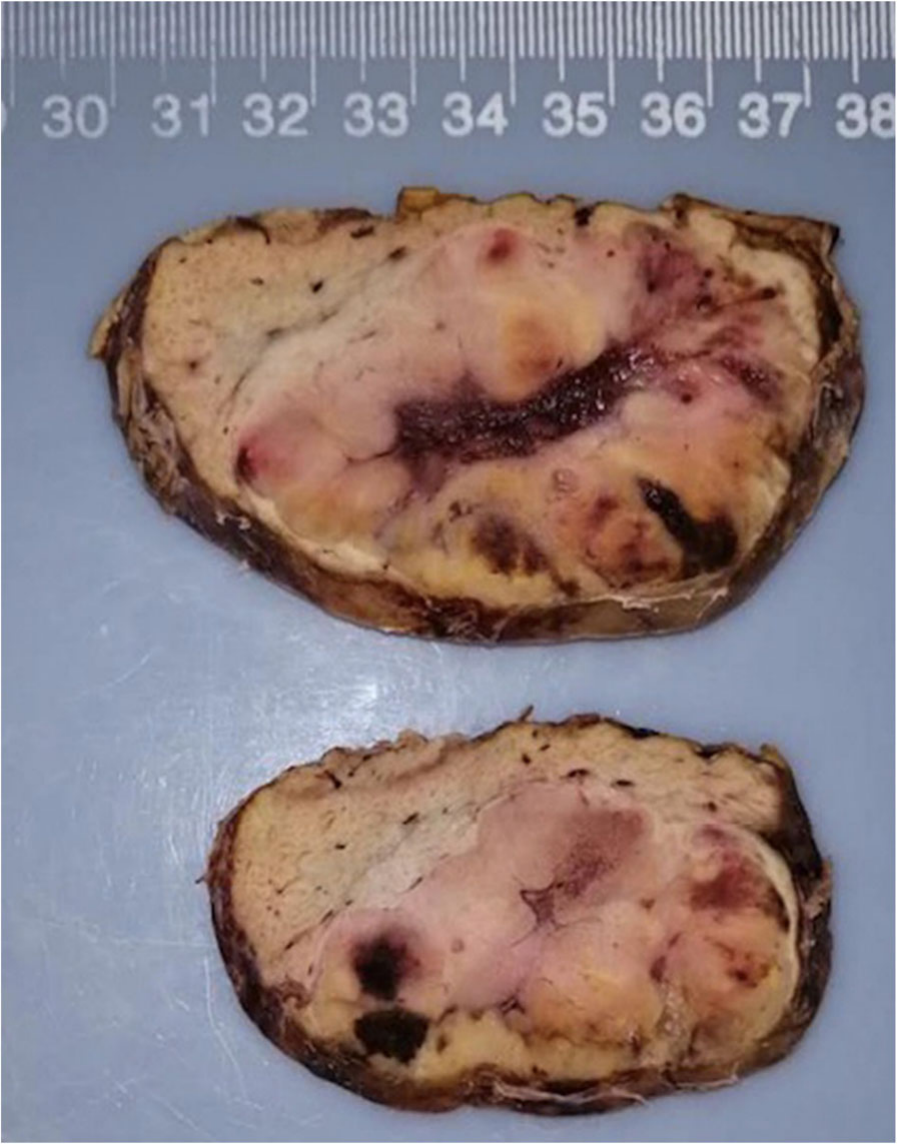
Gross features of the ectopic adrenocortical oncocytic adenoma. The mass had a relatively clear margin with no obvious capsule. The cut surface was yellowish-grey with focal hemorrhage

The specimen measured 8.5 × 8 × 4.5 cm. On the cut surface, a mass measuring 6 × 5.5 × 4 cm was found immediately beneath the liver capsule. The mass had a relatively clear margin with no obvious capsule. Its cut surface was solid and yellowish-grey with focal hemorrhage (Fig. 2). No necrosis was observed. The surrounding liver tissue showed no abnormalities.

**Fig. 3:**
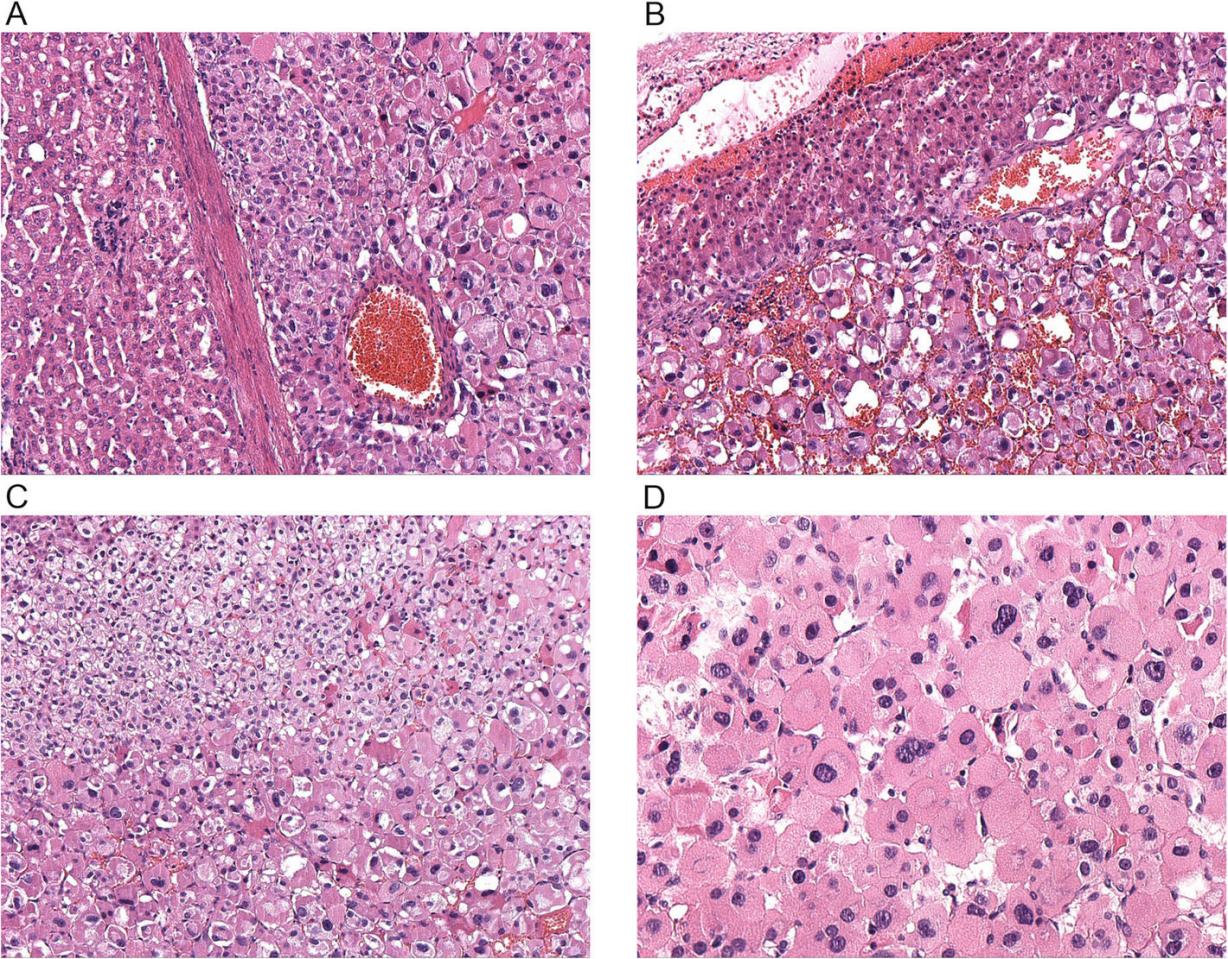
Histopathology findings of the ectopic adrenocortical oncocytic adenoma. **a** The neoplasm was partially encapsulated with a thin capsule (HE x 100). **b** The tumor cells contact directly with hepatocytes with no capsule (HE x 100). **c** Large, eosinophilic polyclonal cells were intermixed with smaller, clear cells mimicking the zona fasciculata of the adrenal gland (HE x 100). **d** Nuclear polymorphism and nucleoli were obvious in eosinophilic cells (HE x 200)

Microscopically, the lesion was incompletely encapsulated (Fig. 3a, b). Cells were arranged in solid sheets and partial hemorrhage could be seen. There was little stroma and no obvious arterialization. Most of the tumor cells were large, polygonal, had prominent nucleoli and were rich in eosinophilic cytoplasm (Fig. 3c). Pleomorphic nucleus was frequently found (Fig. 3d). Nonetheless, the nuclear to cytoplasmic ratio was low. No mitoses, necrosis, or vascular invasion were observed. Focally, smaller cells were found with small nuclei and granular cytoplasm. The surrounding liver tissue was near-normal and the surgical margins were clear. HCC was suspected and perivascular epithelioid tumor (PEComa) was also taken into consideration.

**Fig. 4:**
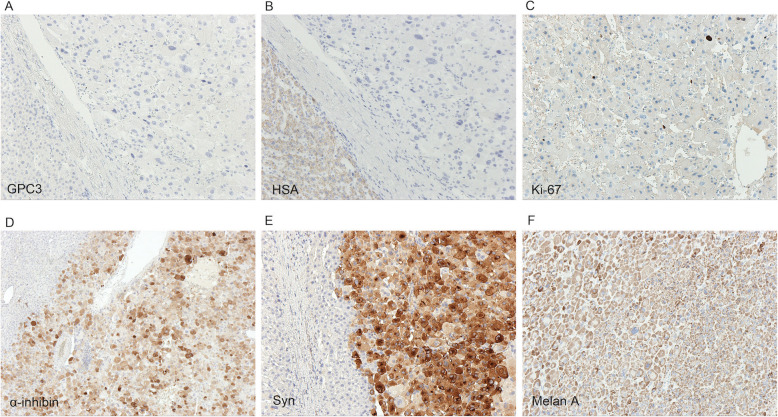
Immunophenotype of the ectopic adrenocortical oncocytic adenoma. **a** Neoplastic cells were negative for GPC3 (IHC x 100). **b** The tumor cells were negative for HSA. Normal hepatocytes (lower left) were used as internal positive control (IHC x 100). **c** The Ki-67 index was 1 % (IHC x 100). **d** α-inhibin was positive in tumor cells and negative in hepatocytes (upper left) (IHC x 100). **e** Syn was positive in tumor cells (right) and negative in hepatocytes (left) (IHC x 100). **f** Melan A was positive in tumor cells (IHC x 100)

Immunohistochemically, the tumor cells were negative for Arg-1, glypican-3 (GPC3), hepatocyte specific antigen (HSA), epithelial membrane antigen (EMA), CK7, CK19, desmin, HMB45, and chromogranin A (CgA). Synaptophysin (Syn), α-inhibin and Melan A were positive. The Ki-67 index was 1 % (Fig. 4). The final diagnosis was ectopic adrenocortical oncocytic adenoma, which was considered to be a benign neoplasm. The patient has been uneventful after the surgery.

## Discussion and conclusions

Ectopic adrenocortical tissue or neoplasms were first described in 1740 and have been most commonly found along the pathway from gonads to adrenal glands [2, 5]. However, sporadic case reports have illustrated that it might manage to gain access in some way and appear in various other sites, including spinal canal, gastric wall, gallbladder, thorax, and liver [3, 6–9]. Intrahepatic adrenocortical neoplasms are extremely rare and their diagnostic challenges have only been discussed from the point of imaging [8, 10–12]. Here, we review the clinicopathological features of ectopic adrenocortical neoplasms that have been misdiagnosed as HCC and discuss the diagnostic pitfalls from the point of clinicians and pathologists.

The clinical features of cases that mimick HCC are summarized in Table 1. Most of the patients are old or middle-aged people, without an obvious sex predilection. All of the neoplasms are located close to the adrenal gland. Radiologically, almost all the cases were diagnosed as HCC before pathological examination, and intrahepatic adrenocortical neoplasm was taken into consideration only in one case (case 7 in Table 1) [13]. The tumors are always nonfunctional.

**Table 1:** Clinical features of cases of intrahepatic adrenocortical neoplasms with diagnostic pitfalls

Case No.	Sex/Age	Tumor location	Tumor function	Manifestations that highly mimick HCC	Diagnosis	Follow-up	Reference
1	F/55	Right lobe	Nonfunctioning	CT	Adrenal rest tumor	N/A	[8]
2	F/66	Adrenohepatic fusion	Nonfunctioning	CT	Adrenocortical adenoma	N/A	[14]
3	M/62	Right lobe	Nonfunctioning	CT	Adrenocortical adenoma	N/A	[15]
4	M/45	Right lobe	Nonfunctioning	CT, MRI, biopsy	Adrenal rest tumor	N/A	[16]
5	F/56	Right lobe	Nonfunctioning	CT and MRI	Oncocytic adrenocortical adenoma	No recurrence for 6 years	[13]
6	M/75	Right lobe	Nonfunctioning	CT	Adrenocortical carcinoma	No recurrence for 2 years	[13]
7	F/64	Adrenohepatic fusion	Nonfunctioning	Biopsy^a^	Adrenocortical adenoma	No recurrence for 4 years	[13]
8	F/59	Adrenohepatic fusion	Nonfunctioning	CT and MRI	Adrenocortical adenoma	No recurrence for 3 years	[12]
Our case	M/44	Right lobe	Nonfunctioning	CT and pathology	Oncocytic adrenocortical adenoma	Currently well	

*M* male; *F* femal; *N/A* not available

^a^The diagnosis for biopsy was hepatocellular carcinoma

Six of the eight previous cases contain detailed pathological description (Table 2). Their pathological features are usually classic and the diagnosis is not difficult as long as this lesion is kept in mind. Nonetheless, in case 4, a biopsy could not differentiate HCC, ectopic adrenal adenoma, and renal cell carcinoma. In case 7, the lesion was misdiagnosed as HCC during biopsy, considering the eosinophilic cytoplasm, nuclear polymorphism, positivity for GPC3, and sinusoidal staining pattern of CD34.

**Table 2:** Pathological characteristics of adrenocortical neoplasms

Case No.	Capsule	Growth pattern	Tumor Cells	Mitotic activity	Other features	IHC
1	Incomplete fibrous capsule	Trabeculae	Clear cells with abundant lipid contents	Low	N/A	N/A
3	N/A	N/A	Clear cells with abundant lipid contents	Low	N/A	N/A
4	Complete fibrous capsule	Sheets or nests	Clear cells with abundant cytoplasm	N/A	N/A	N/A
5	Fibrous capsule	Sheets or vague nodules	Abundant, granular, oncocytic cytoplasm and centrally located round nuclei; inconspicuous nucleoli	N/A	N/A	Positive: α-inhibin, Syn, Melan A
6	Fibrous capsule	Solid sheets and cords	Abundant eosinophilic cytoplasm and a large nucleus with conspicuous nucleoli; occasional nuclear hyperchromasia and moderate cellular pleomorphism	1–2/50HPFs	Evident necrosis; capsule invasion; vascular invasion	Positive: α-inhibin, Melan A Negative: EMA, S-100, CgA, HSA
7	N/A	Sheets	Abundant clear and eosinophilc cytoplasm; mild pleomorphism, enlarged and hyperchromatic nuclei; evident nucleoli; occasional cytoplasmic brownish pigmentation	Low	N/A	Positive: α-inhibin, Syn, Melan A Partial positive: GPC3 Sinusoidal staining pattern: CD34 (in biopsy)

*HPF* high power field

In our case, the lesion was considered to arise primarily from the liver during the surgery. The macroscopic feature of the lesion shows a huge, single distinct nodule with heterogeneity and no obvious capsule, which could certainly happen in HCC. Microscopically, the lesion has a solid growth pattern with high cellularity. The tumor cells seem to show hepatocytic differentiation: Large, eosinophilic tumor cells with striking nucleus polymorphism and prominent nucleoli are characteristic in this case, together with scattered or focal small, clear cells. The lesion was firstly considered to be poorly differentiated hepatocellular carcinoma, with differential diagnosis including metastatic tumors and PEComa. There are several notable points: First, the frequent features in HCC, including combined growth patterns, necrosis, vascular invasion, high mitotic rate, intrahepatic metastasis, are not detected in our case. Second, only a minority of HCC cases develop in a normal or near-normal liver background, especially in elder patients [17, 18]. Third, the patient has carried this lesion for five years with no symptoms, which is incompatible with the fast progression time and poor prognosis of liver cancer. Further IHC studies have demonstrated that the tumors cells are negative for all the hepatocytic markers. Negative staining for HMB45 and desmin almost excludes the possibilities of PEComa. Positive staining for Melan A, α-inhibin, and Syn and negative staining for CgA indicates the differentiation towards adrenal cortex [19, 20]. Based on the low Ki-67 index, few mitoses, and no necrosis or vascular invasion, further review of the slides confirms that the morphology is compatible with adrenocortical adenoma rather than adrenocortical carcinoma. Therefore, our final diagnosis is ectopic adrenocortical oncocytic adenoma.

The present case and review of the literature indicate that there are diagnostic pitfalls before and during pathological examination. Prior to the surgery, there is no method to differentiate an ectopic adrenocortical tissue from HCC, especially when the patient has history of excess alcohol or other risk factors of HCC. Pathologically, when the ectopic adrenocortical adenoma has substantial oncocytic changes, it is easily misdiagnosed as HCC. Several points should be of notification: First, an ectopic adrenocortical neoplasm should be kept in mind, even when the tumor arises from the liver. Second, the arrangement of cells and their characteristics should be carefully examined and factors suggesting malignancy such as necrosis and vascular invasion should be carefully examined. Third, the markers of HCC are not specific enough, and there has been one case in which ectopic adrenocortical adenoma partially expresses GPC3 (Table 2) [13, 21, 22]. Therefore, a combination of HCC markers and adrenocortical markers is strongly suggested. For further confirmation, markers indicating adrenal gland differentiation (α-inhibin, Melan A, and Syn) should be used. Limitation of our work is the lack of investigation into pathogenesis.

The clinical manifestation of this patient and all the previous cases with follow-up information is relatively benign. No evidence of metastasis or recurrence is found, which is consistent with the biological behaviour of adrenocortical adenoma. Currently, our patient is visiting the outpatient regularly and is uneventful.

In conclusion, intrahepatic adrenocortical oncocytic adenoma is an extremely rare lesion which can be easily misdiagnosed as HCC both before surgery and during pathological examination. Our report focuses on providing clues for surgeons and pathologists.

## Data Availability

The dataset supporting the conclusion of this article is included within the article.

## Abbreviations

HCC: Hepatocellular carcinoma
GPC3: Glypican-3
HSA: Hepatocyte specific antigen
EMA: Epithelial membrane antigen
CgA: Chromogranin A
Syn: Synaptophysin
CT: Computed tomography
PEComa: Perivascular epithelioid tumor
